# Measuring the quality of life of patients with diabetic retinopathy in northern India: a comparison of generic and vision specific instruments

**DOI:** 10.1186/s12955-025-02340-8

**Published:** 2025-02-21

**Authors:** Neha Purohit, Aarti Goyal, Vishali Gupta, Parul Chawla Gupta, Prakash Singh, Shankar Prinja

**Affiliations:** 1 Department of Community Medicine, School of Public Health, Post Graduate Institute of Medical Education and Research, Sector-12, Chandigarh, 160012 India; 2 Department of Ophthalmology, Post Graduate Institute of Medical Education and Research, Chandigarh, India

**Keywords:** Diabetic retinopathy, Quality of life, Patient reported health outcomes, EQ-5D-5L, NEI-VFQ-25, Utility values

## Abstract

**Background:**

Estimation of health-related quality of life (HRQoL) in diabetic retinopathy (DR) patients is important to assess the impact of disease, to monitor the treatment outcomes, and conduct health technology assessments. The study aimed to measure the HRQoL in DR patients using the generic as well as disease specific instruments, identify the determinants of HRQoL, empirically investigate the concurrent validity between the QoL instruments, and to develop statistical approaches to map NEI-VFQ-25 (National Eye Institute- Visual Function Questionnaire- 25) composite score based on EQ-5D-5 L (EuroQol 5-dimensions 5-levels) utility values.

**Methods:**

A facility based cross-sectional study was carried out to measure the HRQoL of 300 DR patients using EQ-5D-5 L, EuroQol visual analogue scale (EQ-VAS), and NEI-VFQ-25 instruments. Mean HRQoL scores, along with DR state specific and visual acuity specific utility values were analysed. Pearson correlation coefficient was used to ascertain concurrent validity between NEI-VFQ-25 composite score and its sub-scale scores, EQ-5D-5L index value, and EQ-VAS score. Lastly, we evaluated statistical models using predictor sets based on the EQ-5D-5 L utility scores to estimate NEI-VFQ-25 scores.

**Results:**

The mean EQ-5D-5 L utility value for DR patients was 0.69 (95% CI: 0.65–0.73). The mean NEI-VFQ-25 composite score and EQ-VAS score were 61.1 (95% CI: 57.5–64.5), and 67.6 (95% CI: 65.6–69.5), respectively. Both EQ-5D-5 L and EQ-VAS had a strong concurrent validity with NEI-VFQ-25 composite score. ‘Tobit regression with log of composite score’ was assessed to the preferred model to predict the NEI-VFQ-25 composite score using EQ-5D-5L utility values.

**Conclusion:**

Diabetic retinopathy has a decremental impact on quality of life, which increases with severity in vision loss. Both generic as well as disease-specific outcome measures are equally reliable to determine quality of life of patients with DR. The EQ-5D-5 L can be utilized for measurement of HRQoL in DR in clinical settings to optimize time of clinicians, with further derivation of NEI-VFQ-25 values through application of the crosswalk algorithm for predicting vision- related QoL.

**Supplementary Information:**

The online version contains supplementary material available at 10.1186/s12955-025-02340-8.

## Key messages

*What is known about the topic?*

• There is a dearth of literature on health-related quality of life (HRQoL) in patients with diabetic retinopathy (DR) using generic measures in Indian population.

• EQ-5D-3L depicts weak correlation with NEI-VFQ-25 for ophthalmic disorders, which has led to deliberations on addition of vision bolt-on dimension to the generic tool to increase its validity in measurement of HRQoL. However, the literature lacks assessment of concurrent validity between EQ-5D-5 L and NEI-VFQ-25 tools specifically for DR.

• There have been attempts to map disease specific tools on EQ-5D-3L, however, there is a gap in determination of statistical approaches that can aid in predicting NEI-VFQ-25 scores based on EQ-5D-5L utility values.

*What this study adds?*

• The study informs on valuation and determinants of HRQoL in patients of DR using direct, indirect generic, and disease specific measures- EQ-VAS, EQ-5D-5L, and NEI-VFQ-25 in Indian population.

• There is a strong correlation between generic and disease- specific HRQoL instruments and both are equally reliable to determine QoL in DR patients.

• The model using ‘Tobit regression with log of composite score’ provides the best algorithm to predict NEI-VFQ-25 composite score using EQ-5D-5L utility values.

*How might this study affect research, practice and policy?*

• The study highlights decrement in HRQoL in population with DR. The findings can be used to facilitate health technology assessments and prioritize policies for management of DR. The study calls for strengthening focus on interventions to improve mental health for a responsive management of DR.

• Visual acuity is a strong determinant of quality of life in DR patients. Hence, it is imperative to prioritize preventive strategies, screening and early detection along with treatment to proactively prevent or arrest the progression of vision loss.

• The study highlights that the EQ-5D-5L exhibits sufficient sensitivity to capture the impact of DR on QoL without addition of vision bolt-on dimension. It would be important for the researchers across the world to compare the EQ-5D-5 L with disease-specific tools individually for ophthalmic disorders beyond DR, for decision-making on addition of vision bolt-on dimension to EQ-5D-5L.

• The study findings support the use of EQ-5D-5L, which is relatively cognitively less demanding, and can help optimize the time of clinicians, researchers, as well as the patients. The cross-walk equations can be further used to compute NEI-VFQ-25 composite score, for assessment of vision-related quality of life.

**Supplementary Information:**

The online version contains supplementary material available at 10.1186/s12955-025-02340-8.

## Introduction

Blindness is among the most severe forms of physical disability and has a serious impact on the social and psychological aspects of an individual’s life. India contributes to one-fifth of the world’s blind and visually impaired population [1]. Diabetic retinopathy (DR) has been acknowledged as a significant cause of visual impairment and blindness, and the burden of this disease is likely to rise with advancement of years due to increase in caseload of diabetes [2]. Patient-reported outcomes are recognized as essential measures for the evaluation of medical as well as public health interventions, especially for chronic diseases like DR, where more than survival, the goal of interventions is to improve, or preserve quality of life. The concept of health-related quality of life (HRQoL) not only summarizes impact of the disease but quantifies the effectiveness of health programmes and interventions. Thus, it is a valuable measure to inform on resource allocation decisions, treatment choices, as well as to set priorities for health programmes [3].

Healthcare researchers have used three types of instruments for measuring HRQoL in DR patients - direct, indirect generic, and vision-specific function scales (Online resource material 1). The direct methods, as time trade-off (TTO), standard gamble (SG), and EuroQol visual analogue scale (EQ-VAS), map preferences directly on utility scale and techniques, and have been used to assess the HRQoL in DR [4, 5, 6, 7]. The indirect generic instruments are designed to summarize wide dimensions of the concept of health and can be used in populations across demographic and cultural subgroups, different diseases and treatments. These measures help to make comparisons of health outcomes in different populations and are typically used in health technology assessments. Indirect generic measures, such as, EuroQol five-dimensional three-levels instrument (EQ-5D-3L), Health Utilities Index-3 (HUI-3), 15-dimensional instrument (15-D) and short form 36-dimensional instrument (SF-36), have been used to assign value to quality of life in DR patients [7, 8, 9]. The third type of measure (disease specific scales) which estimate quality of life based on severity of disease symptoms or the visual impact of DR include national eye institute - visual function questionnaire (NEI-VFQ-25), World Health Organization prevention of blindness and deafness vision function-20 (VF-20) questionnaire, retinopathy dependent quality of life (RetDQoL) questionnaire, and audit of diabetes dependent quality of life (ADDQoL) questionnaire [10, 11, 12].

It was interesting to note that barring a few studies in India, Brazil, Taiwan, and Kenya, most of the studies on utility valuation in DR patients have been conducted on populations in the developed nations. There is no gold standard in measuring HRQoL, which has led to use of diverse methodological approaches. In Indian context, six studies on estimation of HRQoL pertaining to DR were identified, out of which, four used validated disease specific instruments (NEI-VFQ-25 questionnaire, ReTDQoL, and ADDQoL questionnaire), while two utilized generic measures (combination of TTO and EQ-5D-3L, and SF health survey) [12, 13, 14, 15]. The existing studies presented their findings with limitations of small sample size and using United Kingdom health state weights for utility calculation. Due to its excellent psychometric properties and ability to allow for comparisons of HRQoL in different disease conditions, EQ-5D-5L has been a preferred for assessing health outcomes for the purpose of health technology assessments in various countries including India [16, 17, 18]. However, none of the Indian studies utilized this measure. Thus, there is dearth of India-specific value set based studies that provide reliable information on QoL of DR patients, using generic as well as disease-specific instruments.

Secondly, despite the inherent dissimilarities between the measures, in practice different instruments are often used interchangeably. It is likely that different measures may provide with different results, and this can create dilemmas and have serious implications while making decisions on resource allocations, and choice of interventions [19]. Since performance of the methods of estimation as well as the relationship between the generic and disease-specific measures depend on the characteristics of the disease states being valued, there is a need to investigate how these measures differ specifically for DR. We specifically aim to assess the correlation between generic EQ-5D-5L tool, and the disease-specific NEI-VFQ-25 tool. EQ-5D-5L tool was chosen for the assessment, since it has been endorsed for HRQoL measurement according to the Indian guidelines for conducting economic evaluations [18]. Furthermore, the Indian value set of EQ-5D-5L instrument for deriving utility values has been published and made available in the public domain, making it the preferred generic instrument [20]. The choice of NEI-VFQ-25 was guided by its use in clinical settings in India and validity of the instrument for assessment of HRQoL in patients with DR [13].

Additionally, it has also been noted that the EQ-5D tool is relatively cognitively undemanding due to the low number and broad scope of the questions and can be administered in a few minutes. On the other hand, the NEI-VFQ-25 tool, which contains 25 questions increases the cognitive load on the patient, as they are required to consider a wider range of scenarios and recall more specific details and it takes about 10 min for the interviewee to elicit responses from the participants [21, 22]. The characteristics of the EQ-5D-5L tool, therefore, make it conducive to be administered to the patients, especially in clinical care settings and in low-resource environments with high patient volumes. However, for evaluating clinical interventions that specifically impact vision-related quality of life, the use of disease-specific tools is recommended [23]. Thus, an optimal approach could involve utilizing the EQ-5D-5L to assess overall HRQoL, followed by mapping the EQ-5D-5L values onto the NEI-VFQ-25 scores to efficiently determine vision-specific QoL. This approach would also allow the health providers to focus on clinical care, rather than on the extensive data collection required for evaluating patient-reported outcomes. However, while there have been attempts to map disease specific tools on EQ-5D, there is a gap in determination of statistical approaches that can aid in predicting NEI-VFQ-25 scores based on EQ-5D utility values [24, 25].

The aim of this study was to measure the HRQoL in patients with DR using the most used indirect, direct generic, and disease specific measures- EQ-5D-5L, EQ-VAS, and NEI-VFQ-25, identify the determinants of HRQoL, empirically investigate the concurrent validity between the three types of instruments, as well as to develop statistical approaches to map NEI-VFQ-25 composite score based on EQ-5D-5L utility values.

## Methodology

### Study design

A facility based cross-sectional study was carried out to measure the quality of life of DR patients. The study involved recruitment of known patients of DR, who visited the ophthalmic outpatient department in a tertiary care hospital of northern India. Diagnosis of DR was based on slit lamp examination and standardized grading of photographs by retinal specialists. Macular edema was confirmed through images obtained from optical coherence tomography, and visual acuity (VA) was assessed with help of Snellen’s chart. Staging of DR was based on the international classification system, recommended by the International Council of Ophthalmology [26]. DR was classified into four stages: mild non-proliferative (NP) DR, moderate NPDR, severe NPDR, and proliferative DR. Diabetic maculopathy was classified as present or absent for all the participants. The definition recommended by the World Health Organization were used for classification of visual acuity [27].

### Sample size and sample selection

A minimum sample size of 277 was calculated considering the reported standard deviation of 0.19 [28], 5% margin of error, 95% confidence intervals, and five categories of visual acuity, and using the formula:

$$\:\text{Sample\:Size\:=\:}{\left(\raisebox{1ex}{$\left({c}_{\alpha\:}*\:\sigma\:\right)$}\!\left/\:\!\raisebox{-1ex}{$MOE$}\right.\right)}^{2}$$,

where MOE is the margin of error (5%), $\:\sigma\:$ is standard deviation and $\:{c}_{\alpha\:}\:$is the critical value at level at 95% confidence intervals.

All the patients above 18 years of age, who were diagnosed with DR by a retinal specialist were eligible to participate in the study, irrespective of their gender or type of diabetes. The exclusion criteria for recruitment included patients aged less than 18 years, and patients who refused to provide written consent for interviews.

### Data collection

Consecutive sampling was done to recruit the sample, with individuals being selected in the order of their presentation to the retina clinic at the outpatient department, contingent upon their eligibility and willingness to participate in the study. Face-to-face interviews were conducted with the eligible participants with DR by appropriately trained research assistant, so as to minimize the response bias in the study. The interviews were conducted with help of a semi-structured questionnaire tool in 2023. The data collection tool comprised of three sections on socio-demographic profile, clinical profile, and quality of life (Online resource material 1). The clinical profile of the participants was validated with help of record review.

### Quality of life tools

The HRQoL was assessed with help of translated local language versions of three types of tools (NEI-VFQ- 25, EQ-5D-5L, and EQ-VAS) (Online resource material 1).

#### EQ-5D-5L

EQ-5D-5L is a standardized generic instrument, comprising of five dimensions: mobility, self-care, usual activities, pain/ discomfort, and anxiety/ depression. Each of these attributes has five levels: no problems, slight problems, moderate problems, severe problems, and extreme problems [21]. The participants were asked about their current level in each of five attributes. We used the tariff values from the reference population set of India to derive the index utility scores [21]. The range of utility scores derived from the Indian value set is -0.923 to + 1.000 [20]. Utility value of “1” denotes perfect health, while “0” indicates QoL equivalent to death, and a negative utility value implies QoL worse than death.

#### EQ-VAS

EQ-VAS is a direct measure of QoL which consists of a 100 mm vertical scale, presenting values between 0 and 100 [29]. The patients were requested to mark the point on the line that best corresponds to their present health state value. The scores represent the ordinal rankings of the health outcomes, where ‘0’ denotes the worst health state and ‘100’ denotes the best health state from the patients’ perspective.

#### NEI-VFQ- 25

The visual function questionnaire is designed to measure the dimensions of vision-targeted health status for persons with chronic eye diseases. It has a base set of 25 questions, running along 11 vision specific domains and one general health domain (Online resource material 1) [22]. Each question has 5 or 6 options, which are recoded later in a range of 0 to 100, where 0 represents the lowest score while 100 is the highest score. Later items within each domain are averaged to create 12 sub-scale scores. The composite score is calculated by taking average of 11 vision-related sub-scale scores and excluding the general health sub-scale. Higher value of the composite score represents a better QoL.

### Statistical analysis

The data were analysed using Statistical Package for Social Sciences (SPSS Inc., Chicago, IL, USA), MS- Excel (Microsoft Corporation, Washington, USA), and STATA version 13 (StatCorp, College Station, TX, USA). Descriptive analyses were undertaken to assess the socio-demographic and clinical characteristics of the sample and reported in terms of frequencies/ proportions of the sample. The mean HRQoL and standard deviation were calculated for all categories of socio-demographic and clinical characteristics related variables, using the NEI-VFQ-25 scores, EQ-5D-5L values, and EQ-VAS scores. DR stage specific and vision specific mean utility scores were also calculated descriptively. The utility values for each descriptive health state using EQ-5D-5L was derived using the Indian EQ-5D-5L value set [20].

Multivariable regression was used to assess the determinants of HRQoL scores (EQ-5D-5 L utility score, EQ-VAS score and NEI-VFQ-25 composite scores), individually. The multiple linear model was:

1 $$\:Y={b}_{0}+{b}_{1}{X}_{1}+{b}_{2}{X}_{2}+\dots\:+{b}_{k}{X}_{k}+e$$

where Y is the outcome variable, $\:{X}_{i}$ is the value of the i^th^ predictor, and e is the error. We used utility score (VAS score and composite scores) as a dependent variable, while the socio-demographic and clinical parameters of patients were used as predictors to understand their influence on the response variable. Normality of error term for all models was checked using Kolmogorov Smirnov Test, with not statistically significant *p*-values as 0.051 (EQ-5D-5L utility score), 0.274 (EQ-VAS score) and 0.067, (NEI-VFQ-25 composite score). The presence of homoscedasticity was checked using Breusch-Pagan Test, with statistically significant *p*-values as 0.028 (EQ-5D-5L utility score), 0.005 (EQ-VAS score) and 0.001, (NEI-VFQ-25 composite score), which reject the null hypothesis of homoscedasticity. Thus, the assumptions of normality of the error term were fulfilled but assumption of presence of homoscedasticity was violated for all the models. There is no multicollinearity with variance inflation values in between 1 and 1.61. Thus, to avoid biased and inconsistent estimates, we used a generalized linear model method using maximum likelihood estimation approach for estimation of associations [30]. This approach relaxes the assumptions of normality of response and homoscedasticity and provides consistent estimators for further use. The relationship in the generalized linear model is assumed to be

2 $$\:Y=g({b}_{0}+{b}_{1}{X}_{1}+{b}_{2}{X}_{2}+\dots\:{b}_{k}{X}_{k})+e$$

where $\:{X}_{i}$ is the value of the *i*^th^ predictor, *e* is the error, and $\:g\left(\right)$ is a function. Formally, the inverse function of $\:g\left(\right)$, say $\:f\left(\right)$, is called the link function; so that

3 $$\:f\left( {{\mu _y}} \right) = {b_0} + {b_1}{X_1} + {b_2}{X_2} + \ldots \:{b_k}{X_k}$$

where $\:{\mu\:}_{y}$ stands for the expected value of *y*. Based on the distribution of the 𝑌 variable, we used identity link function for the current analysis [31, 32].

Concurrent validity was ascertained using the Pearson correlation coefficient (r) to compare the NEI-VFQ-25 composite score and its sub-scale scores, EQ-5D-5L index value, and EQ-VAS score. Validity was also analysed according to the health states of DR and presenting visual acuity. The strength of the relationship was classified according to Evan’s classification and considered very weak (*r* < 0.20), weak (*r* = 0.20–0.39), moderate (*r* = 0.40–0.59), strong (0.60–0.79) and very strong (0.8-1) [33].

As the subsequent phase, we examined various statistical models employing predictor sets derived from EQ-5D-5 L utility scores to estimate NEI-VFQ-25 composite score using the STATA version 13. Firstly, backward stepwise regression was used to inform the choice of independent variables to include in final models, since it has shown to outperform other stepwise selection procedures. The significance level for removal from the model was set at 0.2. The best subset of independent variables was selected using the coefficient of determination *R*^*2*^. Subsequently, four statistical modelling techniques were explored, categorized into linear regression using ordinary least square (OLS) method and Tobit regression, with and without log transformation. We used the OLS and the tobit model for mapping, since they are the most used and recommended models for deriving mapping functions [25, 34]. OLS is the base model which estimates the relationship between independent and continuous dependent variables by minimizing the sum of squared residuals. Secondly, the tobit model is tailored for censored data, where the dependent variable is only observed within a specific range (as in this case, the value of NEI-VFQ-25 lies between 0 and 100). Therefore, Tobit model offers a potential advantage over OLS and similar methods. The Tobit model assumes that NEI-VFQ-25 is a linear function of the covariates plus a residual, and the residuals are normally distributed and homoscedastic (i.e. of equal variance). Further, the log transformation of these models helped stabilize variance and achieve a more normal distribution, improving the assumptions of regression analysis.

The NEI-VFQ-25 composite score was the dependent variable while EQ-5D-5L utility value was the independent variable, for derivation of the mapping function. Model performance was gauged using metrics such as Akaike Information Criteria (AIC), Bayesian Information Criteria (BIC), root mean squared error (RMSE) and mean absolute error (MAE). Based on the outcomes of AIC, BIC, RMSE, and MAE, the model with the superior performance (i.e., exhibiting the lowest AIC, BIC, RMSE, and MAE) was chosen and subjected to further evaluation through regression analyses, considering the significance of predictor variables. Pearson’s correlation coefficient was used to assess the validity between the predicted and observed NEI-VFQ-25 composite score.

## Results

### Sample characteristics

A total of 300 participants were recruited for the study. The response rate was 98.4%. Around 60% of the patients were in the 40–59 years age group, and 62% were males. Almost half of the patients were from the rural areas (51%) and were employed (51%). Most of the recruited patients were known cases of type II diabetes (96%), had a controlled glycaemic status (80.5%), and presented in late stages of PDR (44%) or PDR along with macular oedema (36%). Almost one-sixth (14.7%) of the participants suffered from bilateral blindness while 34.3% had unilateral blindness. Among the study participants, majority of patients had received laser treatment (63%) or combination of laser treatment and intravitreal injections (24%). Detailed demographic and clinical characteristics of the sample are presented in Tables 1 and 2 respectively.

**Table 1:** Socio-demographic details and mean quality of life scores in patients with diabetic retinopathy in different socio-demographic groups

Demographic factors	Number of patients *n*(%)	Mean EQ-5D-5 L score (SD)	Mean NEI-VFQ-25 composite score (SD)	Mean EQ-VAS score (SD)
**Age (years)**				
< 40	23 (7.7%)	0.75 (0.26)	61.35 (27.47)	66.96 (18.45)
40–59	179 (59.7%)	0.70 (0.36)	62.65 (32.14)	68.44 (17.68)
> 60	98 (32.7%)	0.67 (0.3)	58.15 (29.49)	66.12 (14.9)
**Gender**				
Male	185 (61.67%)	0.70 (0.33)	61.97 (30.5)	67.68 (17.65)
Female	115 (38.33%)	0.69 (0.33)	59.65 (31.7)	67.39 (15.56)
**Area**				
Rural	153 (51%)	0.73 (0.28)	59.8 (29.5)	67.39 (16.08)
Urban	147 (49%)	0.66 (0.37)	62.41 (32.4)	67.76 (17.68)
**Education**				
Illiterate	55 (18.33%)	0.70 (0.28)	57.14 (31.15)	66.09 (14.39)
Literate	245 (81.67%)	0.69 (0.34)	61.96 (30.88)	67.9 (17.37)
**Employment**				
Employed	154 (51.33%)	0.74 (0.29)	63.67 (30.53)	69.03 (17)
Unemployed	146 (48.67%)	0.65 (0.36)	58.35 (31.22)	66.03 (16.63)
**Caste**				
General	181 (60.3%)	0.71 (0.32)	63.19 (30.65)	68.59 (16.88)
Non-general	119 (39.7%)	0.68 (0.35)	57.88 (31.21)	66.01 (16.77)
**Religion**				
Hindu	181 (60.3%)	0.68 (0.34)	59.61 (31.04)	66.93 (17.0)
Sikh	104 (34.7%)	0.70 (0.33)	62.62 (30.81)	68.27 (17.01)
Others	15 (5.0%)	0.81 (0.2)	68.11 (31.07)	70.33 (14.45)
**Wealth quintile**				
Poorest	90 (30%)	0.71 (0.31)	60.66 (29.64)	66.67 (16.21)
Poor	50 (16.67%)	0.72 (0.31)	65.34 (29.14)	69.7 (14.96)
Middle	44 (14.67%)	0.65 (0.39)	59.76 (30.9)	66.82 (16.6)
Rich	85 (28.33%)	0.69 (0.31)	56.32 (31.97)	67.29 (17.47)
Richest	31 (10.33%)	0.70 (0.41)	70.37 (33.58)	68.55 (20.62)

**Table 2:** Clinical characteristics and mean quality of life scores in diabetic retinopathy patients with different clinical profile

Clinical factors	Number of patients *n*(%)	Mean EQ-5D-5 L score (SD)	Mean NEI-VFQ-25 composite score (SD)	Mean EQ-VAS score (SD)
**Type of diabetes**				
Type I	12 (4%)	0.70 (0.32)	57.71 (29.9)	62.92 (20.05)
Type II	288 (96%)	0.70 (0.33)	61.22 (31.02)	67.76 (16.72)
**Duration of diabetes**				
≤ 10 years	146 (48.67%)	0.71 (0.33)	60.28 (30.45)	69.01 (16.26)
11–15 years	53 (17.67%)	0.75 (0.3)	69.05 (31.1)	71.32 (16.03)
> 15 years	101 (33.67%)	0.66 (0.35)	58.06 (31.14)	63.51 (17.49)
**Glycaemic control**				
Controlled	239 (80.47%)	0.71 (0.33)	62.78 (30.97)	68.47 (16.3)
Uncontrolled	58 (19.53%)	0.64 (0.36)	53.69 (30.71)	63.88 (18.78)
**Duration of DR**				
1–6 months	220 (73.33%)	0.69 (0.33)	61.23 (30.67)	67.27 (16.39)
7–12 months	50 (16.67%)	0.73 (0.3)	62.26 (30.88)	68.8 (18)
> 12 months	30 (10%)	0.66 (0.42)	58.06 (33.72)	67.67 (18.74)
**Stage of DR** **(Worse eye)**				
NPDR	31 (10.33%)	0.82 (0.21)	66.67 (29.22)	74.35 (14.82)
PDR	133 (44.33%)	0.72 (0.33)	63.52 (30.32)	68.16 (16.63)
NPDR + ME	29 (9.67%)	0.72 (0.2)	58.61 (30.77)	68.62 (15.17)
PDR + ME	107 (35.67%)	0.62 (0.37)	57.09 (32.06)	64.58 (17.63)
**Number of affected eyes**				
1	16 (5.3%)	0.68 (0.35)	60.52 (32.11)	68.13 (17.21)
2	284 (94.7%)	0.70 (0.33)	61.11 (30.92)	67.54 (16.87)
**Visual acuity (Better eye)**				
Normal	77 (25.67%)	0.92 (0.11)	89.02 (9.65)	80.32 (8.36)
Mild	61 (20.33%)	0.85 (0.14)	82.52 (16.89)	76.31 (10.32)
Moderate	85 (28.33%)	0.72 (0.24)	56.31 (20.66)	66.88 (12.58)
Severe	33 (11%)	0.42 (0.43)	28.52 (14.17)	54.09 (11.56)
Blind	44 (14.67%)	0.27 (0.31)	16.1 (11.36)	44.55 (14.58)
**Treatment status**				
Laser	190 (63.3%)	0.68 (0.34)	58.94 (31.54)	66.76 (16.79)
Intravitreal injections and laser	72 (24%)	0.71 (0.34)	63.38 (31.01)	66.94 (17.49)
Others	12 (4%)	0.70 (0.44)	71.29 (20.8)	78.33 (15.86)
Not on treatment	26 (8.7%)	0.77 (0.17)	65.64 (29.68)	70.19 (14.8)
**Co-morbidities**				
Yes	41 (13.67%)	0.61 (0.41)	61.6 (29.29)	67.8 (17.1)
No	259 (86.33%)	0.71 (0.32)	61 (31.24)	67.53 (16.85)

### Quality of life scores

**Fig. 1:**
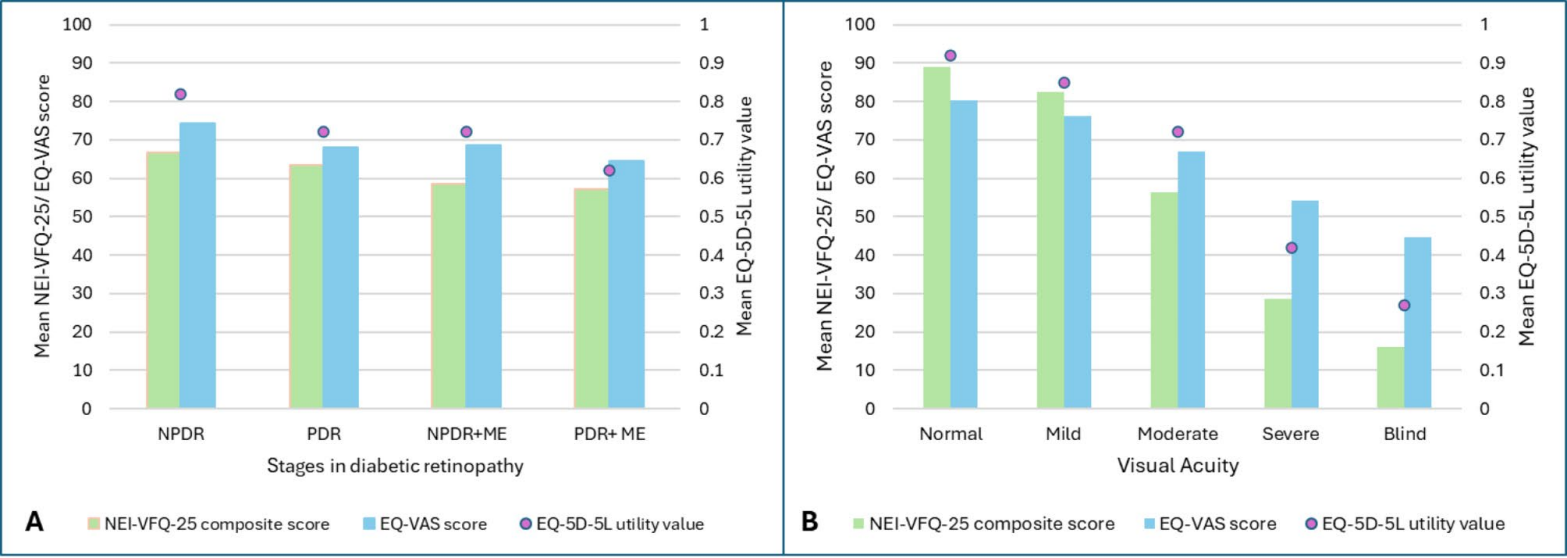
Mean NEI-VFQ-25 scores, EQ-5D-5 L utility value, and EQ-VAS score according to different DR stages **(A)** and level of visual acuity **(B)**

The mean EQ-5D-5L utility score was estimated as 0.69 (95% CI: 0.65–0.73). The mean NEI-VFQ-25 composite score and EQ-VAS score were 61.1 (95% CI: 57.5–64.5), and 67.6 (95% CI: 65.6–69.5), respectively. The HRQoL in patients with different socio-demographic and clinical characteristics is detailed in Tables 1 and 2. According to the EQ-5D instrument, the most reported problem by the patients of DR was anxiety/depression (91%), followed by difficulties in performing usual activities (58%) and mobility (56%). According to NEI-VFQ-25 questionnaire, least scores were obtained for domains of driving difficulties (39.12), followed by issues in near vision activities (51.47), difficulties in general vision (52.47), general health (53.58), role difficulties (57.04), and dependency (57.53). PDR was significantly associated with lower EQ-5D index value (0.72) and EQ-VAS score (68), as compared to NPDR state. The occurrence of PDR along with macular oedema was associated with the lowest HRQoL value according to the three instruments (Table 2; Fig. 1A). A declining trend of HRQoL values was observed in related to decrement in visual acuity (Fig. 1B).

### Determinants of health-related quality of life

It was analysed that after controlling for all the dependent variables, visual acuity was significantly associated with HRQoL assessed from all the three instruments (Table 3). The trend of decreasing utility values with worsening visual acuity (*p* < 0.001) support the construct validity of the instruments. In addition, EQ-5D-5L scores were significantly better for participants residing in rural areas, females, employed population, and in DR patients without macular edema, while EQ-VAS scores varied significantly with duration of diabetes.

**Table 3:** Determinants of quality of life in patients with diabetic retinopathy

		EQ-5D Utility Value			EQ-VAS Score			NEI-VFQ-25 Composite Score		
		Coef.	*p*-value	95% CIs	Coef.	*p*-value	95% CIs	Coef.	*p*-value	95% CIs
**Area**	Rural	**0.085**	0.005	(0.026, 0.145)	-0.624	0.678	(-2.317, 3.564)	-1.171	0.564	(-5.143, 2.802)
**Gender**	Female	**0.094**	0.039	(0.005, 0.183)	3.010	0.180	(-1.391, 7.412)	4.630	0.127	(-1.315, 10.576)
**Employment status**	Employed	**0.134**	0.002	(0.050, 0.219)	3.404	0.111	(-0.777, 7.586)	3.678	0.202	(-1.970, 9.326)
**Duration of Diabetes**	11–15 years	0.033	0.393	(-0.043, 0.110)	1.087	0.558	(-2.556, 4.73)	4.717	0.070	(-0.382, 9.816)
	> 15 years	-0.013	0.685	(-0.078, 0.052)	**-4.209**	0.010	(-7.411, -1.006)	-0.337	0.879	(-4.663, 3.989)
**Stage of DR**	NPDR	0.080	0.111	(-0.018, 0.178)	3.291	0.183	(-1.555, 8.137)	-0.374	0.911	(-6.919, 6.172)
	PDR	**0.070**	0.027	(0.008, 0.132)	1.018	0.514	(-2.037, 4.073)	3.001	0.154	(-1.126, 7.128)
	NPDR + ME	0.061	0.234	(-0.040, 0.162)	1.098	0.665	(-3.874, 6.070)	-1.490	0.664	(-8.206, 5.226)
**Visual acuity better eye**	Mild	-0.011	0.795	(-0.094, 0.072)	-2.686	0.197	(-6.762, 1.390)	-2.640	0.347	(-8.146, 2.866)
	Moderate	**-0.175**	0.000	(-0.251, -0.099)	**-12.472**	0.000	(-16.222, -8.723)	**-29.765**	0.000	(-34.830, -24,701)
	Severe	**-0.469**	0.000	(-0.566, -0.373)	**-25.196**	0.000	(-29.967, -20.425)	**-60.031**	0.000	(-66.475, -53.587)
	Blind	**-0.637**	0.000	(-0.727, -0.547)	**-34.949**	0.000	(-39.370, -30.527)	**-71.688**	0.000	(-77.660, -65.716)
**Co-morbidities**	No	**-0.140**	0.001	(-0.222, -0.058)	-2.407	0.243	(-6.450, 1.637)	-4.700	0.092	(-10.162, 0.762)
**Constant**		**0.706**	0.000	(0.497, 0.916)	**72.006**	0.000	(61.687, 82.326)	**86.310**	0.000	(79.085, 93.535)
**R square**		0.563			0.568			0.774		
**Adjusted R square**		0.522			0.548			0.757		

Bold numbers imply significance at *p*-value < 0.05. The detailed table with results from multiple linear regression and generalized linear regression is present in online resource material, as Table S1 and Table S2

### Concurrent validity of quality-of-life instruments

**Fig. 2:**
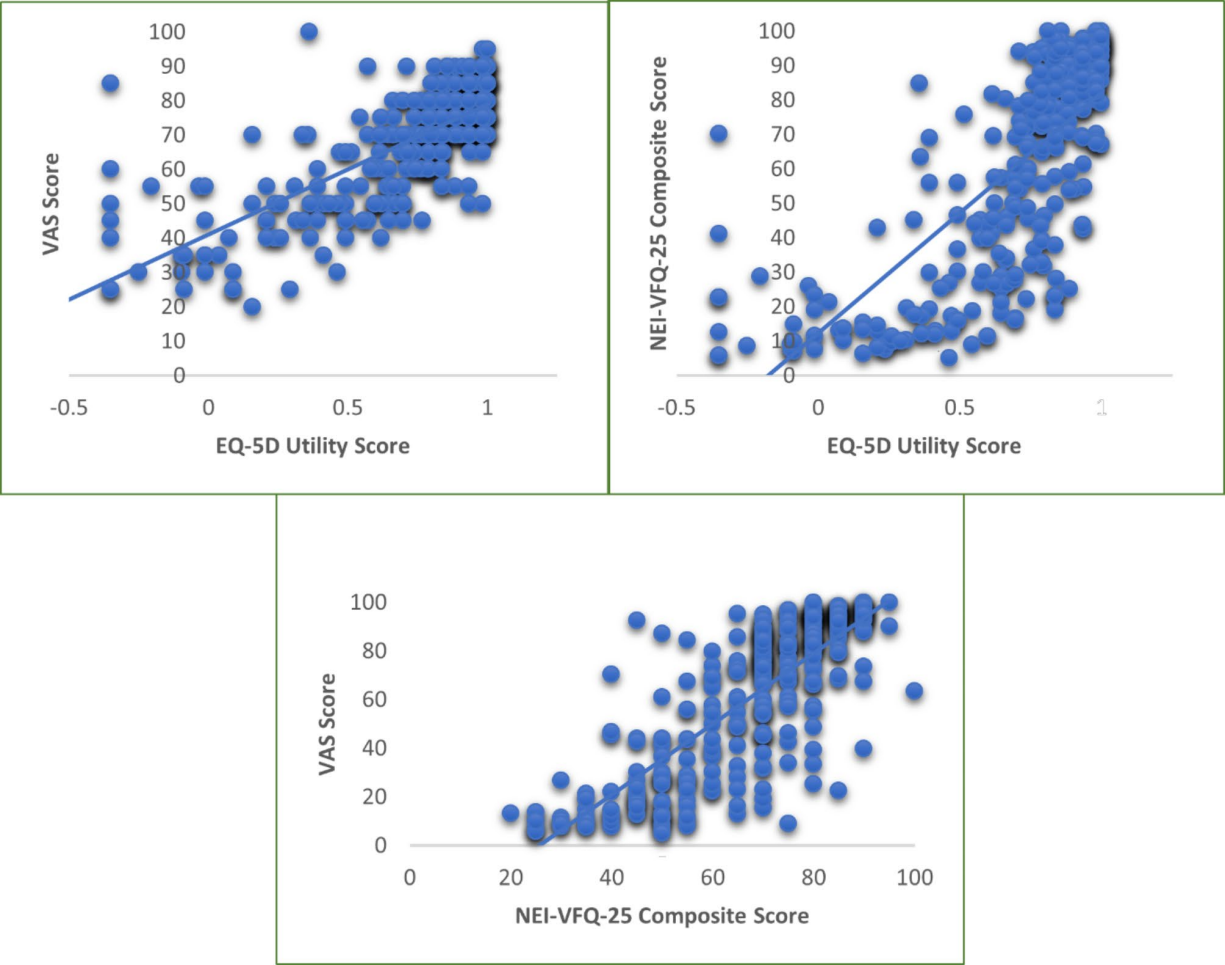
Correlation between quality-of-life instruments in context of diabetic retinopathy

EQ-5D-5L showed a strong significant concurrent validity when compared to EQ-VAS score (*r* = 0.745, *p*-value < 0.001) as well as the NEI-VFQ-25 score (*r* = 0.749, *p*-value < 0.001), and the sub-scale scores of NEI-VFQ-25 questionnaire (Fig. 2, Table S3). It had the highest concurrent validity with domains of ‘difficulties in distance vision activities’, ‘social functioning’, and ‘peripheral vision’. The correlation of VAS score with NEI-VFQ-25 and most of its sub-scales was also found to be strong. The correlation between EQ-5D-5L value and VAS score was least (moderate) for the sub-scale of ‘ocular pain’.

The analysis of the stage wise concurrent validity between the instruments informed that EQ-5D-5L had strong concurrent validity with NEI-VFQ-25 in all stages of DR. Similarly, VAS score was found to be strongly correlated with NEI-VFQ-25 score for all the health states in DR (Table S4). However, the generic instruments had a moderate to strong agreement with the disease specific instrument values in mild, moderate, and blind stages of visual impairment (Table S5). There was no significant correlation between the utility values and composite scores in severe visual impairment and no visual impairment states.

### Mapping model results for crosswalk between EQ-5D and NEI-VFQ-25

The stepwise backward regression informed that among all the socio-demographic and clinical factors, visual acuity in the better eye was the only significant independent variable influencing the relationship between HRQoL derived from EQ-5D-5L and NEI-VFQ-25. The mapping models were developed using the EQ-5D-5L utilities and visual acuity in better eye as independent variable and NEI-VFQ-25 as dependent variable.

All of the models prepared to predict the NEI-VFQ-25 composite score using EQ-5D-5L utility value generated statistically significant coefficients (Table 4). Model 1 A- ‘Tobit with log of NEI-VFQ-25 composite score (Eq. 2) was preferred over the other three models, because it has the smallest AIC (188.5), BIC (214.5), MAE (0.2058) and RMSE (0.3195). Additionally, in model 1A, the data fulfilled the underlying assumptions of multicollinearity, homoscedasticity, and normality of the error term.

4 $$\begin{gathered}\:Composite\:Score = \hfill \\\,\,\,\,\,{e^{3.680 + 0.852*Utility - 0.352*\left( {Moderate\:VA} \right) - 0.751*\left( {Severe\:VA} \right) - 1.198*\left( {Blind} \right)}} \hfill \\ \end{gathered} $$

The correlation coefficient of observed and predicted score for NEI-VFQ-25 composite score was 0.899, which indicated very strong concurrence. The predicted NEI-VFQ-25 composite scores for 3125 EQ-5D-5L health states are provided in online resource material 2.

**Table 4:** Model performance for predicting NEI-VFQ-25 composite score based on EQ-5D-5 L utility values

		Model 1 A Tobit Regression with Log of Composite Score			Model 2 A Tobit Regression			Model 3 A OLS with log of Composite Score			Model 4 A OLS		
		Coef.	*p*-value	95% CIs	Coef.	*p*-value	95% CIs	Coef.	*p*-value	95% CIs	Coef.	*p*-value	95% CIs
Utility value		**0.852**	0.000	(0.698, 1.005)	**28.356**	0.000	(21.7, 35.01)	**0.842**	0.000	(0.688, 0.996)	**28.435**	0.000	(21.73, 35.14)
Vision in better eye	Mild	-0.041	0.457	(-0.15, 0.068)	-4.570	0.058	(-9.3, 0.16)	-0.042	0.451	(-0.151, 0.068)	-4.565	0.061	(-9.33, 0.2)
	Moderate	**-0.352**	0.000	(-0.456, -0.248)	**-27.045**	0.000	(-31.57, -22.52)	**-0.354**	0.000	(-0.458, -0.249)	**-27.029**	0.000	(-31.59, -22.47)
	Severe	**-0.751**	0.000	(-0.903, -0.599)	**-46.385**	0.000	(-52.99, -39.78)	**-0.756**	0.000	(-0.909, -0.603)	**-46.346**	0.000	(-53, -39.69)
	Blind	**-1.198**	0.000	(-1.354, -1.043)	**-54.687**	0.000	(-61.45, -47.93)	**-1.199**	0.000	(-1.355, -1.042)	**-54.479**	0.000	(-61.28, -47.68)
Constant		**3.680**	0.000	(3.521, 3.838)	**63.027**	0.000	(56.17, 69.88)	**3.689**	0.000	(3.53, 3.847)	**62.954**	0.000	(56.05, 69.86)
R Square		0.735			0.164			0.8012			0.7966		
Adjusted R Square								0.7978			0.7931		
AIC		188.55			2441.5			179.0656			2443.744		
BIC		214.57			2467.426			201.2883			2465.966		
MAE		0.2058			10.26743			0.207			10.27025		
RMSE (95% CI)		0.3195 (-0.0035, 0.037)			13.92907 (-1.562, 1.608)			0.3197 (-0.036, 0.036)			13.92893 (-1.585, 1.585)		
Breuch- Pagan								0.0510			0.0883		
VIF		1.44–2.27											

Bold number signify significance at *p*-value < 0.001

Coeff: Coefficient, CI: Confidence Intervals, OLS: Ordinary Least Square, AIC: Akaike Information Criteria, BIC: Bayesian Information Criteria, MAE: Mean Absolute Error, RMSE: Root Mean Squared Error, VIF: Variance Inflation Factor

## Discussion

In pursuit of patient-centred and cost-effective healthcare, the relevance of HRQoL is growing, in terms of defining the standard of care. We determined the utility values for different health states associated with DR in a sample of patients using three types of QoL measures. The NEI-VFQ-25 composite score and general health score was estimated to be 60.43 and 53.58, which was similar to the scores reported by previous studies on DR patients conducted in the southern part of India (Table S6) [13, 14, 35, 36, 37]. Next, we consider that this is the first study from India to measure HRQoL values in DR patients using EQ-5D-5 L instrument, and hence it was not possible to validate the findings with the existing literature from India. However, the estimated mean utility score of 0.69 from the study, was similar to that reported by Zare et al. [38] in Iran using EQ-5D-5L questionnaire, but lower than the value (0.97) reported by Pan et al. [39] in China (Table S6). This could be attributed to a number of differences: variations in study settings, clinical characteristics of the sample, cultural variations, and different value-sets used to compute the EQ-5D-5L utility values [39].

Considering the HRQoL of general population in India as 0.848 [40], a decrement of 0.158 in utility value was noted in patients with DR, which calls for policies to ensure prevention and early detection of the disease. This utility value is important for the researchers conducting heath technology assessments related to DR. The findings further suggested that the co-existence of macular edema with PDR was associated with a significant decrement in HRQoL when compared to EQ-5D-5L utility value in patient with PDR, however there was non-significant declining trend in state wise QoL values with disease progression, according to EQ-VAS and NEI-VFQ-25 questionnaire. Previous studies using EQ-5D-5L instrument have also reported a significant declining gradient in self-reported QoL with increasing severity of the disease [28, 41]. Similar to our findings, Das et al. (2016) highlighted the non-significant difference in NPDR and PDR state related HRQoL derived from NEI-VFQ-25 in Indian DR patients [41].

We found no independent association between utility scores and socio-demographic characteristics except for the area of residence and gender. Our findings of a significant association of QoL with gender is similar to those reported by Pawar et al. [13], although contrasting to the finding reported by Polack et al. and Pereira et al. [14, 15]. Additionally, the population residing in rural areas reported significantly higher HRQoL, in concordance with the findings by Kamran et al. in Iranian patients with DR [42]. While this finding may be counterintuitive due to better access to healthcare in urban areas, it is not implausible in Indian context. In rural areas, stronger familial and social support systems, along with a relatively lower emphasis on the significance of ailments compared to urban areas, could contribute to a pronounced positive impact on overall well-being. It was also noted that QoL was not related to type of diabetes and glycaemic control in the patients. This lack of association has been described in the several research studies [14, 28, 35]. The presence of co-morbidities led to a significant decrement in HRQoL derived from EQ-5D-5L, similar to the findings by Polack et al. [15]. The results informed that declining vision in diabetes was associated with substantial reduction in utility, according to the generic as well as disease-specific instruments. This was in concurrence with the existing literature, which informs that severity of vision loss is a very significant determinant of QoL, and in lines with the general biological plausibility [35]. Hence, it is imperative to prioritize preventive strategies, screening and early detection along with treatment to proactively prevent or arrest the progression of vision loss.

As reported in our study, the earlier studies on population with DR have similarly highlighted that sub-scales of ocular pain, colour vision and social functioning present with relatively higher scores, while general health, general vision, and driving have been associated with lower scores compared to other domains of NEI-VFQ-25 [14, 43, 44] (Online Resource Material 1). Both the instruments used in the study highlighted the impact of the disease on mental health. This was in consensus with the existent research studies, which describe depression as the most common problem in DR patients [45, 46]. This implied that interventions aimed at improving HRQoL in DR patients should focus on enhancing mental health through counselling, social support programs, and education to help patients cope with the emotional challenges of living with a chronic condition. Specifically in India, the comprehensive primary healthcare programme is being leveraged to provide a basket of diverse services inclusive of ophthalmic care as well as mental healthcare to the population through the primary healthcare teams [47]. Such health initiatives can help reduce anxiety and depression in patients with DR, leading to a more holistic and patient-centred approach to care.

Our study suggested a strong correlation of EQ-5D-5L and EQ-VAS instruments with NEI-VFQ-25, and this association remained consistent when disease states were considered. Additionally, EQ-5D-5L and EQ-VAS presented strong correlation, highlighting the internal consistency of the participant responses. We also found moderate to strong agreement between HRQoL values derived from the generic and disease specific tools in mild, moderate, and blind stages of visual impairment. However, lack of significant correlation in severe visual impairment could be attributed to low sample of patients in this group. Earlier studies have reported a fair correlation between NEI-VFQ-25 and EQ-5D/ EQ-VAS and suggested the insensitivity of generic QoL instruments in visual conditions [9, 48]. However, these studies had utilized EQ-5D-3L instrument instead of more updated and sensitive version of EQ-5D-5L [49].

There has been deliberation regarding the potential increase in sensitivity of EQ-5D-5L through the addition of a “bolt-on” vision dimension [50, 51]. However, the incorporation of additional vision dimension may alter valuation for the five EQ-5D-5L dimensions. Hence, the absence of a well-established valuation for each additional bolt-on dimension raises concerns about the potential compromise in comparability between studies incorporating bolt-ons and those without any. Furthermore, the introduction of a vision bolt-on may impact the correlation between visual impairment and other co-morbidities [52]. The EQ-5D-5L demonstrated adequate sensitivity to capture the HRQoL in DR patients in the study. However, it would be too premature to oppose the inclusion of vision bolt-on to generic instrument based on these findings, as this warrants further research into the validity of the EQ-5D-5L in other ophthalmic conditions before advocating for the inclusion of a vision-specific bolt-on to the generic instrument. It is plausible that the EQ-5D-5L, in its current form, is sufficiently sensitive to assess HRQoL in chronic conditions like DR, which have a significant effect on general health beyond their impact on vision, unlike other eye diseases that may have a more limited effect on general health. Given the potential for differential impacts across ophthalmic disorders, it is likely that these conditions would affect both the subscales of the NEI-VFQ-25 and the dimensions of the EQ-5D-5L in distinct ways, depending on the nature of the disease. Therefore, the correlation between the EQ-5D-5L and NEI-VFQ-25, should be evaluated on a case-by-case basis for each ophthalmic disorder before making a broader decision regarding the inclusion of a vision-specific bolt-on to the EQ-5D-5L tool.

The crosswalk analysis suggested that ‘Tobit with log of composite score’ model performed better than models based on alternative distributions, to identify the best algorithm for prediction of NEI-VFQ-25 composite score. The strong predictive power of the mapping equations further supports its use for estimation of HRQoL with an acceptable precision. Patient reported outcome measures are increasingly being used in clinical trials as well as routine clinical practice, and the main advantage from this algorithm mapping is that the estimation of NEI-VFQ-25 scores is based on EQ-5D-5L tool interviews, which are less time consuming for both the interviewer and the respondents. This is essentially beneficial in the clinical settings in India and other countries, which present a high patient load, as it allows the physicians to spend a higher time on the treatment than that on the measurement of patient-reported outcomes. However, the mapping function requires assessment in DR population in diverse settings to ensure the robustness and generalizability of the algorithm. Furthermore, such algorithm may be adapted for use in other eye disorders following a careful assessment of the correlation between the instruments, along with a thorough examination of socio-demographic and clinical variables that could influence the relationship between the NEI-VFQ-25 and EQ-5D-5L derived HRQoL values for each ophthalmic disorder.

Our study was limited to a set of population visiting a tertiary care hospital which caters to population from one-fifth of the Indian states. Therefore, the findings of HRQoL may not be generalizable to the entire population of the country. However, the sample was reflective of a broad range of sociodemographic characteristics, and we assessed the HRQoL according to different socio-demographic characteristics. Secondly, to make the crosswalk algorithm generalizable to other settings, we included the significant co-variates influencing the relationship between HRQoL derived from EQ-5D-5L and NEI-VFQ-25. Therefore, there are no strong reasons for believing that this should alter the reported functional relationships between the instruments. We did not use any visual support for administering EQ-VAS, as use of such mode could have resulted in bias due to participants presenting with different visual acuity in the sample. Lastly, since the study was of cross-sectional nature, it has the limitation to assess the responsiveness (sensitivity to change over time) of the instruments to reflect on the objective changes in the constructs, such as severity of disease, over time. Therefore, longitudinal studies are required to measure the impact of clinical interventions/ progression of disease on HRQoL. It would be further valuable to extend the validation of the mapping functions using external data, to ensure the generalizability of the algorithm.

## Conclusion

Diabetic retinopathy has a decremental impact on quality of life, which increases with severity of the disease. The study suggested that generic as well as disease-specific outcome measures are equally useful and reliable to determine quality of life of patients with DR. It offers a deeper knowledge in terms of relationship between EQ-5D-5L and the vision specific instrument and establishing a statistical mapping algorithm for predicting NEI-VFQ-25 score based on the utility values. The findings from the study can be used to highlight the magnitude of impact of DR on quality of life, to inform on policy decisions on strategies for diagnosis and management of DR, as well as to optimize the time of clinicians and researchers in measurement of DR related quality of life. The study calls for future research on addition of vision bolt-on to EQ-5D-5L in individual ophthalmic disorders.

## Electronic supplementary material

Below is the link to the electronic supplementary material.

Supplementary Material 1

Supplementary Material 2

## Data Availability

The datasets used in the current study are available from the corresponding author on reasonable request and after permission of Institute Collaborative Research Committee, Post Graduate Institute of Medical Education and Research, Chandigarh.

## References

[1] GBD 2019 Blindness and Vision Impairment Collaborators, & Vision Loss Expert Group of the Global Burden of Disease Study. Causes of blindness and vision impairment in 2020 and trends over 30 years, and prevalence of avoidable blindness in relation to VISION 2020: the right to Sight: an analysis for the global burden of Disease Study. The Lancet. Global Health. 2021;9(2):e144–60. 10.1016/S2214-109X(20)30489-7. 33275949 10.1016/S2214-109X(20)30489-7 PMC7820391

[2] Raman R, Vasconcelos JC, Rajalakshmi R, Prevost AT, Ramasamy K, Mohan V, Mohan D, Rani PK, Conroy D, Das T, Sivaprasad S, SMART India Study Collaborators. Prevalence of diabetic retinopathy in India stratified by known and undiagnosed diabetes, urban-rural locations, and socioeconomic indices: results from the SMART India population-based cross-sectional screening study. Lancet Global Health. 2022;10(12):e1764–73. 10.1016/S2214-109X(22)00411-9. 36327997 10.1016/S2214-109X(22)00411-9

[3] Deshpande PR, Rajan S, Sudeepthi BL, Abdul Nazir CP. Patient-reported outcomes: a new era in clinical research. Perspect Clin Res. 2011;2(4):137–44. 10.4103/2229-3485.86879. 22145124 10.4103/2229-3485.86879 PMC3227331

[4] Szabo SM, Beusterien KM, Pleil AM, Wirostko B, Potter MJ, Tildesley H, Gonder J, Barsdorf A, Levy AR. Patient preferences for diabetic retinopathy health States. Investig Ophthalmol Vis Sci. 2010;51(7):3387–94. 10.1167/iovs.09-4194. 20053977 10.1167/iovs.09-4194

[5] Tung TH, Chen SJ, Lee FL, Liu JH, Lin CH, Chou P. A community-based study for the utility values associated with diabetic retinopathy among type 2 diabetics in Kinmen, Taiwan. Diabetes research and clinical practice. 2005;68(3):265–73. 10.1016/j.diabres.2004.10.003 10.1016/j.diabres.2004.10.003 15936470

[6] Shah VA, Gupta SK, Shah KV, Vinjamaram S, Chalam KV. TTO utility scores measure quality of life in patients with visual morbidity due to diabetic retinopathy or ARMD. Ophthalmic Epidemiol. 2004;11(1):43–51. 10.1076/opep.11.1.43.26436. 14977496 10.1076/opep.11.1.43.26436

[7] Lloyd A, Nafees B, Gavriel S, Rousculp MD, Boye KS, Ahmad A. Health utility values associated with diabetic retinopathy. Diabet Medicine: J Br Diabet Association. 2008;25(5):618–24. 10.1111/j.1464-5491.2008.02430.x. 10.1111/j.1464-5491.2008.02430.x 18346157

[8] Kontodimopoulos N, Pappa E, Chadjiapostolou Z, Arvanitaki E, Papadopoulos AA, Niakas D. Comparing the sensitivity of EQ-5D, SF-6D and 15D utilities to the specific effect of diabetic complications. Eur J Health Economics: HEPAC: Health Econ Prev care. 2012;13(1):111–20. 10.1007/s10198-010-0290-y. 10.1007/s10198-010-0290-y 21132558

[9] Heintz E, Wiréhn AB, Peebo BB, Rosenqvist U, Levin LÅ. QALY weights for diabetic retinopathy–a comparison of health state valuations with HUI-3, EQ-5D, EQ-VAS, and TTO. Value Health: J Int Soc Pharmacoeconomics Outcomes Res. 2012;15(3):475–84. 10.1016/j.jval.2011.11.031. 10.1016/j.jval.2011.11.031 22583458

[10] Roberts-Martínez Aguirre I, Rodríguez-Fernández P, González-Santos J, Aguirre-Juaristi N, Alonso-Santander N, Mielgo-Ayuso J, González-Bernal JJ. Exploring the Quality of Life Related to Health and Vision in a Group of patients with Diabetic Retinopathy. Healthc (Basel Switzerland). 2–022;10(1):142. 10.3390/healthcare10010142. 10.3390/healthcare10010142 PMC8775606 35052305

[11] Emade N, Nyamori J, Njuguna M, Njambi L, Gichuhi S. Vision-Related Quality of Life among patients attending the diabetes and Eye Clinics in Kenyatta National Hospital, Kenya. J Ophthalmol. 2023;7809692. 10.1155/2023/7809692. 10.1155/2023/7809692 PMC9873415 36703703

[12] Deswal J, Narang S, Gupta N, Jinagal J, Sindhu M. To study the impact of diabetic retinopathy on quality of life in Indian diabetic patients. Indian J Ophthalmol. 2020;68(5):848–53. 10.4103/ijo.IJO_1553_19. 32317460 10.4103/ijo.IJO_1553_19 PMC7350471

[13] Pawar S, Parkar A, Menon S, Desai N, Namrata D, Dole K. Assessment of quality of life of the patients with diabetic retinopathy using National Eye Institute Visual Functioning Questionnaire (VFQ-25). J Healthc Qual Res. 2021;36(4):225–30. 10.1016/j.jhqr.2021.02.004. 33820745 10.1016/j.jhqr.2021.02.004

[14] Pereira DM, Shah A, D’Souza M, Simon P, George T, D’Souza N, Suresh S, Baliga MS. Quality of life in people with Diabetic Retinopathy: Indian study. J Clin Diagn Research: JCDR. 2017;11(4):NC01–6. 10.7860/JCDR/2017/24496.9686. 10.7860/JCDR/2017/24496.9686 PMC5449823 28571177

[15] Polack S, Alavi Y, Rachapalle Reddi S, Kulothungan V, Kuper H. Utility values associated with diabetic retinopathy in Chennai, India. Ophthalmic Epidemiol. 2015;22(1):20–7. 10.3109/09286586.2014.885057. 24669801 10.3109/09286586.2014.885057

[16] Kangwanrattanakul K, Krägeloh CU. EQ-5D-3L and EQ-5D-5L population norms for Thailand. BMC Public Health. 2024;24(1). 10.1186/s12889-024-18391-3.20. 10.1186/s12889-024-18391-3 PMC11036570 38649833

[17] Feng YS, Kohlmann T, Janssen MF, Buchholz I. Psychometric properties of the EQ-5D-5L: a systematic review of the literature. Qual Life Res. 2021;30(3):647–73. 10.1007/s11136-020-02688-y21. 33284428 10.1007/s11136-020-02688-y PMC7952346

[18] Sharma D, Prinja S, Aggarwal AK, Rajsekar K, Bahuguna P. Development of the Indian reference case for undertaking economic evaluation for health technology assessment. Lancet Reg Health Southeast Asia. 2023;16:100241. 10.1016/j.lansea.2023.100241. PMID: 37694178; PMCID: PMC10485782. 37694178 10.1016/j.lansea.2023.100241 PMC10485782

[19] Arnold D, Girling A, Stevens A, Lilford R. Comparison of direct and indirect methods of estimating health state utilities for resource allocation: review and empirical analysis. BMJ (Clinical Res ed). 2009;339:b2688. 10.1136/bmj.b2688. 10.1136/bmj.b2688 PMC2714630 22128393

[20] Jyani G, Sharma A, Prinja S, Kar SS, Trivedi M, Patro BK, Goyal A, Purba FD, Finch AP, Rajsekar K, Raman S, Stolk E, Kaur M. Development of an EQ-5D value set for India using an Extended Design (DEVINE) study: the Indian 5-Level version EQ-5D Value Set. Value Health: J Int Soc Pharmacoeconomics Outcomes Res. 2022;25(7):1218–26. 10.1016/j.jval.2021.11.1370. 10.1016/j.jval.2021.11.1370 35779943

[21] EuroQol Research Foundation. EQ-5D-5L User Guide. 2009. Retrieved September 26, 2024, from: https://euroqol.org/publications/user-guides

[22] National Eye Institute. Visual Function Questionnaire- 25. 2000 version. National Eye Institute. United States of America. 2000. Retrieved September 26, 2024, from: nei.nih.gov/learn-about-eye-health/outreach-resources/outreach-materials/visual-function-questionnaire-25, last assessed on 10.08.2023.

[23] Gnanasakthy A, DeMuro CR. The Limitations of EQ-5D as a Clinical Outcome Assessment Tool. Patient. 2024;17(3):215–217. 10.1007/s40271-024-00683-w. Epub 2024 Mar 11. PMID: 38466537. 10.1007/s40271-024-00683-w 38466537

[24] Payakachat N, Summers KH, Pleil AM, Murawski MM, Thomas J, Jennings K, Anderson JG, Predicting. EQ-5D utility scores from the 25-item National Eye Institute Vision Function Questionnaire (NEI-VFQ 25) in patients with age-related macular degeneration. Qual life Research: Int J Qual life Aspects Treat care Rehabilitation. 2009;18(7):801–13. 10.1007/s11136-009-9499-6. 10.1007/s11136-009-9499-6 19543808

[25] Kay S, Ferreira A. Mapping the 25-item National Eye Institute Visual Functioning Questionnaire (NEI VFQ-25) to EQ-5D utility scores. Ophthalmic Epidemiol. 2014;21(2):66–78. 10.3109/09286586.2014.888456. 24568628 10.3109/09286586.2014.888456

[26] Indian Institute of Public Health, Hyderabad. Guidelines for the Prevention and Management of Diabetic Retinopathy and Diabetic Eye Disease in India. 2019. Hyderabad, India: Indian Institute of Public Health. Retrieved September 26, 2024 from: https://phfi.org/wp-content/uploads/2019/09/2019-Guidelines-for-the-Prevention-and-Management-of-Diabetic-Retinopathy.pdf

[27] World Health Organization. Blindness and visual impairment. Retrieved September 26. 2024 from: https://www.who.int/news-room/fact-sheets/detail/blindness-and-visual-impairment#:~:text=Mild%20%E2%80%93%20visual%20acuity%20worse%20than,acuity%20worse%20than%203%2F60

[28] Ben ÂJ, de Souza CF, Locatelli F, Rosses APO, Szortika A, de Araujo AL, de Carvalho G, Lavinsky D, Neyeloff JL, Neumann CR. Health-related quality of life associated with diabetic retinopathy in patients at a public primary care service in southern Brazil. Arch Endocrinol Metab. 2021;64(5):575–83. 10.20945/2359-3997000000223. 34033298 10.20945/2359-3997000000223 PMC10118975

[29] Feng Y, Parkin D, Devlin NJ. Assessing the performance of the EQ-VAS in the NHS PROMs programme. Quality of life research. Int J Qual life Aspects Treat care Rehabilitation. 2014;23(3):977–89. 10.1007/s11136-013-0537-z. 10.1007/s11136-013-0537-z PMC4287662 24081873

[30] Damodar N. Basic econometrics. The Mc-Graw Hill. 2004.

[31] McCullagh P. Generalized linear models. Routledge; 2019.

[32] Wood SN. Generalized additive models: an introduction with R. Chapman and Hall/CRC. 2017.

[33] Papageorgiou SN. On correlation coefficients and their interpretation. J Orthodont. 2022;49(3):359–61. 10.1177/14653125221076142. 10.1177/14653125221076142 PMC9420886 36017900

[34] Sun Q, Zhang F. Current status of Research on the mapping function of Health Utility values in the Asia Pacific Region: a systematic review. Value Health Reg Issues. 2021;24:224–39. Epub 2021 Apr 21. PMID: 33894684. 33894684 10.1016/j.vhri.2020.12.008

[35] Çetin EN, Bulgu Y, Zencir M, Avunduk AM, Yaylali V, Yildirim C. Vision related quality of life in patients with diabetic retinopathy. J Retina-Vitreous. 2012;20(3):213–7.

[36] Cusick M, SanGiovanni JP, Chew EY, Csaky KG, Hall-Shimel K, Reed GF, Caruso RC, Ferris FL 3. Central visual function and the NEI-VFQ-25 near and distance activities subscale scores in people with type 1 and 2 diabetes. Am J Ophthalmol. 2005;139(6):1042–50. 10.1016/j.ajo.2005.01.008. 15953434 10.1016/j.ajo.2005.01.008

[37] Scanlon PH, Loftus J, Starita C, Stratton IM. The use of weighted health-related quality of Life scores in people with diabetic macular oedema at baseline in a randomized clinical trial. Diabet Medicine: J Br Diabet Association. 2015;32(1):97–101. 10.1111/dme.12593. 10.1111/dme.12593 PMC4307641 25251842

[38] Zare F, Ameri H, Madadizadeh F, Reza Aghaei M. Health-related quality of life and its associated factors in patients with type 2 diabetes mellitus. SAGE open Med. 2020;8:2050312120965314. 10.1177/2050312120965314. 33996077 10.1177/2050312120965314 PMC8107944

[39] Pan CW, Wang S, Wang P, Xu CL, Song E. Diabetic retinopathy and health-related quality of life among Chinese with known type 2 diabetes mellitus. Qual life Research: Int J Qual life Aspects Treat care Rehabilitation. 2018;27(8):2087–93. 10.1007/s11136-018-1876-6. 10.1007/s11136-018-1876-6 29740784

[40] Jyani G, Prinja S, Garg B, Kaur M, Grover S, Sharma A, Goyal A. Health-related quality of life among Indian population: the EQ-5D population norms for India. J Global Health. 2023;13:04018. 10.7189/jogh.13.04018. 10.7189/jogh.13.04018 PMC9936451 36799239

[41] Das T, Wallang B, Semwal P, Basu S, Padhi TR, Ali MH. Changing clinical presentation, current knowledge-Attitude-Practice, and current vision related Quality of Life in Self-reported type 2 diabetes patients with retinopathy in Eastern India: the LVPEI Eye and Diabetes Study. J Ophthalmol. 2016;3423814. 10.1155/2016/3423814. 10.1155/2016/3423814 PMC5098084 27843643

[42] Kamran JS, Jafroudi S, Leili EK, Chafjiri AS, Paryad E. Quality of life in patients with diabetic retinopathy. J Holist Nurs Midwifery. 2017;27(1):69–77.

[43] Kovac B, Vukosavljevic M, Djokic Kovac J, Resan M, Trajkovic G, Jankovic J, Smiljanic M, Grgurevic A. Validation and cross-cultural adaptation of the National Eye Institute Visual Function Questionnaire (NEI VFQ-25) in Serbian patients. Health Qual Life Outcomes. 2015;13:142. 10.1186/s12955-015-0330-5. 26370558 10.1186/s12955-015-0330-5 PMC4570616

[44] Lloyd AJ, Loftus J, Turner M, Lai G, Pleil A. Psychometric validation of the visual function Questionnaire-25 in patients with diabetic macular edema. Health Qual Life Outcomes. 2013;11:10. 10.1186/1477-7525-11-10. 23347793 10.1186/1477-7525-11-10 PMC3599421

[45] Gabrielian A, Hariprasad SM, Jager RD, Green JL, Mieler WF. The utility of visual function questionnaire in the assessment of the impact of diabetic retinopathy on vision-related quality of life. Eye. 2010;24(1):29–35. 10.1038/eye.2009.56. 19325572 10.1038/eye.2009.56

[46] Khoo K, Man REK, Rees G, Gupta P, Lamoureux EL, Fenwick EK. The relationship between diabetic retinopathy and psychosocial functioning: a systematic review. Qual life Research: Int J Qual life Aspects Treat care Rehabilitation. 2019;28(8):2017–39. 10.1007/s11136-019-02165-1. 10.1007/s11136-019-02165-1 30879245

[47] Ministry of Health and Family Welfare. Government of India. Ayushman Bharat Comprehensive Primary Health care through Health and Wellness Centres. Operational Guidelines. 2018. Available from: https://nhsrcindia.org/sites/default/files/2021-03/Operational%20Guidelines%20For%20Comprehensive%20Primary%20Health%20Care%20through%20Health%20and%20Wellness%20Centers.pdf, last accessed on 13.01.2024.

[48] Fenwick EK, Xie J, Ratcliffe J, Pesudovs K, Finger RP, Wong TY, Lamoureux EL. The impact of diabetic retinopathy and diabetic macular edema on health-related quality of life in type 1 and type 2 diabetes. Investig Ophthalmol Vis Sci. 2012;53(2):677–84. 10.1167/iovs.11-8992. 22205611 10.1167/iovs.11-8992

[49] Janssen MF, Bonsel GJ, Luo N, Is. EQ-5D-5L Better Than EQ-5D-3L? A Head-to-Head Comparison of Descriptive Systems and Value sets from Seven Countries. PharmacoEconomics. 2018;36(6):675–97. 10.1007/s40273-018-0623-8. 29470821 10.1007/s40273-018-0623-8 PMC5954015

[50] Longworth L, Yang Y, Young T, Mulhern B, Alava MH, Mukura C, Rowen D, Tosh J, Tsuchiya A, Evans P, Keetharuth AD, Brazier J. Developing ‘Bolt-on’ items to EQ-5D. Southampton: NIHR Journals Library; 2014.

[51] Gandhi M, Ang M, Teo K, Wong CW, Wei YC, Tan RL, Janssen MF, Luo N. A vision ‘bolt-on’ increases the responsiveness of EQ-5D: preliminary evidence from a study of cataract surgery. Eur J Health Economics: HEPAC: Health Econ Prev care. 2020;21(4):501–11. 10.1007/s10198-019-01156-w. 10.1007/s10198-019-01156-w 31902023

[52] Purola PKM, Koskinen SVP, Uusitalo HMT. Comparison of three health-related quality of life instruments in relation to visual acuity: EQ-5D, 15D, and EUROHIS-QOL8. Quality of life research. Int J Qual life Aspects Treat care Rehabilitation. 2023;32(2):543–52. 10.1007/s11136-022-03293-x. 10.1007/s11136-022-03293-x PMC9911483 36385360

