# Supplementary material for: Measuring the quality of life of patients with diabetic retinopathy in northern India: a comparison of generic and vision specific instruments
Source: Health Qual Life Outcomes. 2025 Feb 21;23:17. doi: 10.1186/s12955-025-02340-8 (PMC11846259; doi:10.1186/s12955-025-02340-8)
Supplement: Supplementary file 1 — Supplementary Material 1 [file 12955_2025_2340_MOESM1_ESM.docx]

**Supplementary Material**

# **Measuring the Quality of life of patients with diabetic retinopathy in northern India: A comparison of generic and vision specific instruments**

Contents

[**Measuring the Quality of life of patients with diabetic retinopathy in northern India: A comparison of generic and vision specific instruments** 1](#_Toc187699124)

[Section 1. Details of generic and disease-specific instruments 1](#_Toc187699125)

[Section 2. Details of the semi-structured data collection tool 2](#_Toc187699126)

[A. Socio-demographic profile 2](#_Toc187699127)

[B. Clinical profile 3](#_Toc187699128)

[C. Quality of life tools 3](#_Toc187699129)

[NEI-VFQ-25 3](#_Toc187699131)

[EQ-5D-5L 3](#_Toc187699132)

[EQ-VAS 4](#_Toc187699133)

[**Section 3.** Determinants of quality of life in patients with diabetic retinopathy using different models 5](#_Toc187699134)

[Table S1. Determinants of quality of life in patients with diabetic retinopathy using multiple linear regression using least square method 5](#_Toc187699135)

[Table S2. Determinants of quality of life in patients with diabetic retinopathy using generalized linear regression 6](#_Toc187699136)

[**Section 4.** Concurrent validity between the HRQoL instruments in diabetic retinopathy patients 7](#_Toc187699137)

[Table S3. Concurrent validity of EQ-5D-5L, NEI-VFQ-25, and EQ-VAS in diabetic retinopathy patients 7](#_Toc187699138)

[Table S4. Health state wise concurrent validity of EQ-5D-5L, NEI-VFQ-25, and EQ-VAS in diabetic retinopathy patients 8](#_Toc187699139)

[Table S5. Visual acuity based concurrent validity of EQ-5D-5L, NEI-VFQ-25, and VAS in diabetic retinopathy patients 9](#_Toc187699140)

[**Section 5.** Comparison of HRQoL findings with existing literature 10](#_Toc187699141)

[Table S6. Comparison of existing NEI-VFQ-25 scores with existing literature [5-9] 10](#_Toc187699142)

[Table S7. Comparison of mean EQ-5D value and VAS scores with existing literature [9-15] 11](#_Toc187699143)

[**References** 12](#_Toc187699144)

# Section 1. Details of generic and disease-specific instruments

Health-related quality of life (HRQoL) instruments are essential tools for assessing the impact of health conditions on individuals' well-being, and they can be broadly categorized into generic and disease-specific measures. Generic HRQoL instruments are versatile and can be used across a wide range of populations, enabling comparisons between different diseases, health statuses, and general populations. Their broad applicability makes them useful in large-scale studies and cost-effective for epidemiological research. However, these instruments may lack the sensitivity to detect the unique impacts of specific diseases, potentially missing crucial disease-related aspects that affect quality of life.

In contrast, disease-specific HRQoL instruments are tailored to capture the detailed impact of a particular condition on an individual's health, making them highly sensitive and relevant for monitoring disease progression and assessing treatment outcomes. They provide deeper insights into the unique symptoms and challenges associated with a specific disease, which is crucial for patient care. The downside is that these instruments are limited in generalizability and can’t easily be compared across different health conditions. Additionally, they often come with greater participant burden, as they may be more complex and longer, especially for patients with multiple conditions. Disease-specific measures can also be expensive to develop and may not be readily available for all diseases. In practice, a combination of both generic and disease-specific instruments is often used to leverage the strengths of each, ensuring a more comprehensive understanding of an individual's health and quality of life.

Generic quality of life (QoL) instruments can be further categorized into direct and indirect methods. Indirect generic QoL instruments, such as the EQ-5D or SF-36, ask individuals to assess their own health across various dimensions, including physical, emotional, and social well-being. These instruments are patient-centred and provide a broad, comprehensive view of an individual's health. They are generally easy to use and suitable for a wide range of populations. However, their reliance on self-reporting introduces subjectivity, which can be influenced by personal biases or varying interpretations of questions. Additionally, while indirect instruments offer a holistic view, they may lack the sensitivity to detect subtle changes in health or provide insights into the specific disease-specific factors underlying poor quality of life. On the other hand, direct generic QoL instruments, like the time trade-off (TTO) or standard gamble (SG), involve hypothetical scenarios where individuals make decisions about trading life years for health improvements. These tools offer more standardized, objective measures of health-related quality of life. However, these methods can be complex and difficult for some individuals to understand, especially those with low health literacy. Furthermore, because they are based on hypothetical situations, the results may not always reflect real-life health experiences.

# Section 2. Details of the semi-structured data collection tool

The data collection tool comprised of three sections on socio-demographic profile, clinical profile, and quality of life.

## A. Socio-demographic profile

The data collection tool consisted of questions regarding the participant's current age, gender, area of residence, highest educational qualification, employment status, religion, caste, marital status, health insurance status, and the annual income of the household. Age was recorded in completed years. Gender and area of residence were categorized into binary options: male/female and urban/rural, respectively. The response categories for highest educational qualification, employment status, religion, caste, marital status, and health insurance status were aligned with the data collection formats used in national-level surveys in India. The economic status of the participants was assessed based on the annual income of the household. The sample was divided into five income quintiles, based on the annual income data collected through the data collection instrument.

## B. Clinical profile

The clinical profile section of the questionnaire comprised of details on duration of diabetes, blood glucose level according to diagnostic reports within the last one year of the date of interview, and regular intake of medicines for any other disease conditions, to assess the presence of co-morbidities. Adequate glycaemic control was considered, if the participant presented with diagnostic report, stating HbA1c level <6.5%, or RBS/OGTT<200 mg/dl or fasting plasma glucose<126 mg/dl.

Furthermore, details on duration of diabetic retinopathy (in months), number of eye affected (one or both), stage of diabetic retinopathy (Mild NPDR, Moderate NPDR, Severe NPDR, PDR), presence of macular edema (Yes/ No), and the details of visual acuity on the date of interview were acquired. The definition recommended by the World Health Organization was used for classification of visual acuity. The clinical details of the participant were validated from the patient cards to ensure accuracy.

## C. Quality of life tools

### The HRQoL was assessed with help of translated local language versions of three types of tools (NEI-VFQ- 25, EQ-5D-5L, and EQ-VAS).

### NEI-VFQ-25

The NEI-VFQ-25 comprises of 25 questions that cover a “general health” domain and 11 distinct vision-specific domains: “difficulty with general vision”, “ocular pain”, “difficulty with near vision activities”, “difficulty with distance vision activities”, “difficulties in social functioning due to vision”, “mental health issues due to vision”, “role difficulties due to vision”, “dependency on others due to vision”, “driving issues due to vision”, “limitations with colour vision”, and “limitation with peripheral vision”.

The domains and their corresponding questions include:

1. General Vision: This question evaluates the participant's overall perception of their vision, asking about their ability to see clearly and their satisfaction with their vision.
2. Ocular Pain: This domain assesses the level of discomfort or pain related to the eyes, including questions about eye soreness or irritation.
3. Near Vision: This section focuses on how difficulties with close-up vision (e.g., reading or doing close work) affect daily activities.
4. Distance Vision: This item evaluates the participant’s ability to see things clearly at a distance, such as recognizing faces or driving.
5. Social Functioning: This domain explores how vision impairment affects the individual’s social interactions, including participation in social activities and relationships.
6. Mental Health: This section addresses emotional well-being, specifically how vision problems may cause feelings of frustration, depression, or anxiety.
7. Role Difficulties: This question assesses how vision impairment affects the individual’s ability to perform roles in life, such as at work or home.
8. Dependency: This item evaluates the participant’s reliance on others for assistance with tasks due to vision loss.
9. Driving: This domain asks about the ability to drive or the limitations that vision problems cause with driving.
10. Colour Vision: This section focuses on the ability to perceive colours, particularly how visual impairments may affect colour differentiation.
11. Peripheral Vision: This item addresses difficulties in seeing objects in the peripheral vision, which can affect navigation and awareness of the environment.
12. General Health: The final question considers the participant's overall health status, providing a broad context for understanding their visual functioning.

Each question has 5 or 6 options on a Likert scale, which are recoded later in a range of 0 to 100, where 0 represents the lowest score while 100 is the highest score. The composite score is calculated by taking average of 11 vision-related sub-scale scores and excluding the general health sub-scale. Higher value of the composite score represents a better QoL.

### EQ-5D-5L

The **EQ-5D-5L** instrument consists of five questions, each addressing a specific domain, with five response levels. These five dimensions are:

1. **Mobility**: This dimension assesses the participant's ability to move around. The question asks how their mobility is on the date of interview, with response options ranging from "no problems" to "unable to walk."
2. **Self-care**: This item focuses on the individual’s ability to take care of themselves in terms of daily activities such as dressing, washing, and feeding. The response options also range from "no problems" to "unable to wash or dress myself."
3. **Usual activities**: This domain assesses how health problems impact the individual’s ability to perform their usual work, study, housework, or recreational activities. The response categories range from "no problems" to "unable to perform usual activities."
4. **Pain/discomfort**: This question evaluates the level of pain or discomfort the individual has experienced. The five response levels range from "no pain or discomfort" to "extreme pain or discomfort."
5. **Anxiety/depression**: This domain examines the participant’s emotional well-being, specifically focusing on anxiety or depression over the past week. Responses range from "not anxious or depressed" to "extremely anxious or depressed."

Each of these five questions is scored on a five-level scale, with higher levels indicating more severe problems.

### EQ-VAS

EQ-VAS comprises of a single question which asks the participant to rate their general health on a scale from 0 to 100, where 0 represents the "worst health you can imagine" and 100 represents the "best health you can imagine."

# **Section 3.** Determinants of quality of life in patients with diabetic retinopathy using different models

## Table S1. Determinants of quality of life in patients with diabetic retinopathy using multiple linear regression using least square method

## Table S2. Determinants of quality of life in patients with diabetic retinopathy using generalized linear regression

# **Section 4.** Concurrent validity between the HRQoL instruments in diabetic retinopathy patients

## Table S3. Concurrent validity of EQ-5D-5L, NEI-VFQ-25, and EQ-VAS in diabetic retinopathy patients

|  | **EQ-5D Value** | **VAS Score** | **NEI-VFQ-25 Composite Score** | **General Health** | **General Vision** | **Ocular Pain** | **Near Activities** | **Distance Activities** | **Social Functioning** | **Mental Health** | **Role Difficulties** | **Dependency** | **Driving** | **Colour Vision** | **Peripheral Vision** |
| --- | --- | --- | --- | --- | --- | --- | --- | --- | --- | --- | --- | --- | --- | --- | --- |
| **EQ-5D Value** | 1 | .745^**^ | .749^**^ | .674^**^ | .645^**^ | .508^**^ | .696^**^ | .742^**^ | .742^**^ | .671^**^ | .650^**^ | .635^**^ | .640^**^ | .710^**^ | .740^**^ |
| **VAS Score** |  | 1 | .792^**^ | .704^**^ | .744^**^ | .536^**^ | .751^**^ | .755^**^ | .772^**^ | .702^**^ | .676^**^ | .675^**^ | .678^**^ | .772^**^ | .754^**^ |
| **NEI-VFQ-25 Composite Score** |  |  | 1 | .662^**^ | .876^**^ | .603^**^ | .938^**^ | .958^**^ | .957^**^ | .920^**^ | .890^**^ | .906^**^ | .850^**^ | .938^**^ | .948^**^ |
| **General Health** |  |  |  | 1 | .671^**^ | .394^**^ | .625^**^ | .637^**^ | .637^**^ | .611^**^ | .563^**^ | .579^**^ | .512^**^ | .618^**^ | .625^**^ |
| **General Vision** |  |  |  |  | 1 | .468^**^ | .866^**^ | .845^**^ | .824^**^ | .772^**^ | .745^**^ | .730^**^ | .764^**^ | .799^**^ | .827^**^ |
| **Ocular Pain** |  |  |  |  |  | 1 | .483^**^ | .538^**^ | .536^**^ | .575^**^ | .565^**^ | .556^**^ | .517^**^ | .510^**^ | .520^**^ |
| **Near Activities** |  |  |  |  |  |  | 1 | .929^**^ | .907^**^ | .812^**^ | .784^**^ | .809^**^ | .792^**^ | .875^**^ | .896^**^ |
| **Distance Activities** |  |  |  |  |  |  |  | 1 | .953^**^ | .827^**^ | .805^**^ | .819^**^ | .785^**^ | .922^**^ | .946^**^ |
| **Social Functioning** |  |  |  |  |  |  |  |  | 1 | .834^**^ | .784^**^ | .815^**^ | .767^**^ | .962^**^ | .953^**^ |
| **Mental Health** |  |  |  |  |  |  |  |  |  | 1 | .872^**^ | .909^**^ | .734^**^ | .822^**^ | .822^**^ |
| **Role Difficulties** |  |  |  |  |  |  |  |  |  |  | 1 | .842^**^ | .737^**^ | .771^**^ | .783^**^ |
| **Dependency** |  |  |  |  |  |  |  |  |  |  |  | 1 | .748^**^ | .794^**^ | .809^**^ |
| **Driving** |  |  |  |  |  |  |  |  |  |  |  |  | 1 | .773^**^ | .756^**^ |
| **Colour Vision** |  |  |  |  |  |  |  |  |  |  |  |  |  | 1 | .925^**^ |
| **Peripheral Vision** |  |  |  |  |  |  |  |  |  |  |  |  |  |  | 1 |
| ** Correlation is significant at p-value <0.001 level (2-tailed) | | | | | | | | | | | | | | | |

## Table S4. Health state wise concurrent validity of EQ-5D-5L, NEI-VFQ-25, and EQ-VAS in diabetic retinopathy patients

|  | **Correlation co-efficient in different health states** | | | | | | | |
| --- | --- | --- | --- | --- | --- | --- | --- | --- |
|  | **NPDR** | | **PDR** | | **NPDR+ME** | | **PDR+ME** | |
|  | **VAS Score** | **NEI-VFQ-25 Composite Score** | **VAS Score** | **NEI-VFQ-25 Composite Score** | **VAS Score** | **NEI-VFQ-25 Composite Score** | **VAS Score** | **NEI-VFQ-25 Composite Score** |
| **EQ-5D Value** | 0.807** | 0.690** | 0.671** | 0.766** | 0.557** | 0.796** | 0.835** | 0.754** |
| **VAS Score** | 1 | 0.835** | 1 | 0.781** | 1 | 0.788** | 1 | 0.798** |

** Correlation is significant at p-value <0.01 level

NPDR: Non-proliferative diabetic retinopathy, PDR: Proliferative diabetic retinopathy, NPDR+ME: Non-proliferative diabetic retinopathy with macular edema, PDR+ME: Proliferative diabetic retinopathy with macular edema

## Table S5. Visual acuity based concurrent validity of EQ-5D-5L, NEI-VFQ-25, and VAS in diabetic retinopathy patients

|  | **Correlation co-efficient based on visual acuity** | | | | | | | | | | | | | | |
| --- | --- | --- | --- | --- | --- | --- | --- | --- | --- | --- | --- | --- | --- | --- | --- |
| **Worse Eye** | **Better Eye** | | | | | | | | | | | | | | |
|  | **Normal** | | | **Mild** | | | **Moderate** | | | **Severe** | | | **Blind** | | |
|  | **U*C** | **U*V** | **V*C** | **U*C** | **U*V** | **V*C** | **U*C** | **U*V** | **V*C** | **U*C** | **U*V** | **V*C** | **U*C** | **U*V** | **V*C** |
| **Normal** | -0.14 | 0.18 | 0.19 |  | | |  | | |  | | |  | | |
| **Mild** | 0.03 | **0.88** | -0.04 | **0.87** | -0.29 | -0.06 |  |  |  |  |  |  |  |  |  |
| **Moderate** | 0.19 | 0.09 | **0.49** | **0.61** | **0.73** | **0.67** | **0.86** | **0.65** | **0.65** |  |  |  |  |  |  |
| **Severe** | **1** | **-*** | **-*** | 0.96 |  |  | 0.07 | **0.78** | 0.05 | 0.49 | **0.66** | 0.24 |  |  |  |
| **Blind** | **0.74** | **0.48** | **0.47** | **0.63** | **0.78** | **0.72** | **0.58** | **0.58** | **0.47** | 0.23 | 0.17 | **0.43** | **0.50** | **0.7** | **0.58** |

Bold values signify that Correlation is significant p-value ≤0.05 level *Low sample

U: EQ-5D utility value; V: VAS score, C: Composite score; U*C: correlation between utility value and composite score; U*V: correlation between utility value and VAS score; V*C: correlation between VAS score and composite score

# **Section 5.** Comparison of HRQoL findings with existing literature

## Table S6. Comparison of existing NEI-VFQ-25 scores with existing literature [5-9]

|  | Current study (n=300) | Pawar et al (n=149) | Pereira et al (n=123) | Cetin et al (n=93) | Cusick et al (n=170) | Scanlon et al (n=289) |
| --- | --- | --- | --- | --- | --- | --- |
| Place | India | India | India | Turkey | USA | UK |
| General Health | 53.58 | 32.18 | 58.43 | 49.7 | 50 | 41.1 |
| General vision | 52.47 | 25.06 | 63.69 | 51.5 | 72 | 54.3 |
| Ocular pain | 88.38 | 63.78 | 89.1 | 72.3 | 93 | 78.8 |
| Near vision activities | 51.40 | 31.63 | 70.79 | 60.6 | 78 | 57.9 |
| Distance vision activities | 60.33 | 62.45 | 72.68 | 65.7 | 77 | 63.5 |
| Social functioning | 65.00 | 78.25 | 78.69 | 76.8 | 93 | 79.3 |
| Mental Health | 60.52 | 56.25 | 71.71 | 56.9 | 77 | 55.9 |
| Role difficulties | 57.04 | 46.36 | 74.8 | 57.7 | 81 | 54.4 |
| Dependency | 57.53 | 66.31 | 77.17 | 67.5 | 87 | 68.4 |
| Driving | 39.12 | 52.79 | 48.84 | 62.2 | 79 | 67.9 |
| Colour vision | 65.42 | 79.67 | 76.29 | 79.9 | 90 | 86.2 |
| Peripheral vision | 64.92 | 61.96 | 69.58 | 70.1 | 82 | 71.4 |
| Composite score | 60.43 | 54.72 | 73.93 | 65.9 | 82 | 66.2 |

## Table S7. Comparison of mean EQ-5D value and VAS scores with existing literature [9-15]

|  | Sample size  (DR patients) | Place | EQ-5D value | VAS score |
| --- | --- | --- | --- | --- |
| Current study | 300 | India | 0.69 | 67.6 |
| Polack et al | 219 | India | 0.60* |  |
| Zare et al | 299 | Iran | 0.70 | 65 |
| Ben et al | 206 | Brazil | 0.73* | 70 |
| Pan et al | 913 | China | 0.97 |  |
| Zhang et al | 1341 | China | 0.81* | 67.3 |
| Romero-Naranjo et al | 48 | Ecuador | 0.77* | 77.9 |
| Clarke et al | 3192 | UK | 0.77* | 74 |
| Scanlon et al | 289 | UK | 0.74* |  |

*Values determined from EQ-5D-3L

# **References**

Pawar S, Parkar A, Menon S, Desai N, Namrata D, Dole K. Assessment of quality of life of the patients with diabetic retinopathy using National Eye Institute Visual Functioning Questionnaire (VFQ-25). J Healthc Qual Res. 2021;36(4):225-230.

Pereira DM, Shah A, D'Souza M, Simon P, George T, D'Souza N, Suresh S, Baliga MS. Quality of Life in People with Diabetic Retinopathy: Indian Study. J Clin Diagn Res. 2017;11(4):NC01-NC06.

1. Çetin EN, Bulgu Y, Zencir M, Avunduk AM, Yaylali V, Yildirim C Vision related quality of life in patients with diabetic retinopathy. Retina-Vitreus. 2012;20(3):213-217.
2. Cusick M, SanGiovanni JP, Chew EY et al. Central visual function and the NEI-VFQ-25 near and distance activities subscale scores in people with type 1 and type 2 diabetes. Am J Ophthalmol. 2005;139:1042-50
3. Scanlon, PH, Loftus J, Starita C, Stratton I M. The use of weighted health-related Quality of Life scores in people with diabetic macular oedema at baseline in a randomized clinical trial. Diabetic Medicine. 2015; 32 (1): 97-101.

Polack S, Alavi Y, Rachapalle Reddi S, Kulothungan V, Kuper H. Utility values associated with diabetic retinopathy in Chennai, India. Ophthalmic Epidemiol. 2015;22(1):20-7.

1. Ben ÂJ, de Souza CF, Locatelli F, Rosses APO, Szortika A, de Araujo AL, de Carvalho G, Lavinsky D, Neyeloff JL, Neumann CR. Health-related quality of life associated with diabetic retinopathy in patients at a public primary care service in southern Brazil. Arch Endocrinol Metab. 2021;64(5):575-583.
2. Pan CW, Wang S, Wang P, Xu CL, Song E. Diabetic retinopathy and health-related quality of life among Chinese with known type 2 diabetes mellitus. Qual Life Res. 2018;27(8):2087-2093.
3. Zhang Y, Wu J, Chen Y, Shi L. EQ-5D-3L Decrements by Diabetes Complications and Comorbidities in China. Diabetes Ther. 2020;11(4):939-950.
4. Romero-Naranjo F, Espinosa-Uquillas C, Gordillo-Altamirano F, Barrera-Guarderas F. Which Factors may reduce the Health-Related Quality of Life of Ecuadorian Patients with Diabetes? P R Health Sci J. 2019;38(2):102-108.
5. Clarke P, Gray A, Holman R. Estimating utility values for health states of type 2 diabetic patients using the EQ-5D (UKPDS 62). Med Decis Making. 2002;22(4):340-9.
